# Two Cases of Sporadic Amyotrophic Lateral Sclerosis With Contrasting Clinical Phenotypes: Genetic Insights

**DOI:** 10.7759/cureus.56023

**Published:** 2024-03-12

**Authors:** Andrey Frolov, Miguel A Guzman, Ghazala Hayat, John R Martin

**Affiliations:** 1 Center for Anatomical Science and Education, Saint Louis University School of Medicine, Saint Louis, USA; 2 Department of Pathology, Saint Louis University School of Medicine, Saint Louis, USA; 3 Department of Neurology, Saint Louis University School of Medicine, Saint Louis, USA; 4 ALS Center of Excellence, Saint Louis University School of Medicine, Saint Louis, USA

**Keywords:** whole exome sequencing, next generation sequencing, postmortem genetic screening, clinical heterogeneity, amyotrophic lateral sclerosis

## Abstract

Amyotrophic lateral sclerosis (ALS) is a fatal neuromuscular disease that affects individuals of diverse racial and ethnic backgrounds. There is currently no cure for ALS, and the number of efficient disease-modifying drugs for ALS is limited to a few, despite the large number of clinical trials conducted in recent years. The latter could be attributed to the significant heterogeneity of ALS clinical phenotypes even in their familial forms. To address this issue, we conducted postmortem genetic screening of two female patients with sporadic ALS (sALS) and contrasting clinical phenotypes. The results demonstrated that despite their contrasting clinical phenotypes, both patients had rare pathologic/deleterious mutations in five genes: *ACSM5*, *BBS12*, *HLA-DQB1*, *MUC20*, and *OBSCN*, with mutations in three of those genes being identical: *BBS12*, *HLA-DQB1*, and *MUC20*. Additional groups of mutated genes linked to ALS, other neurologic disorders, and ALS-related pathologies were also identified. These data are consistent with a hypothesis that an individual could be primed for ALS via mutations in a specific set of genes not directly linked to ALS. The disease could be initiated by a concerted action of several mutated genes linked to ALS and the disease's clinical phenotype will evolve further through accessory gene mutations associated with other neurological disorders and ALS-related pathologies.

## Introduction

Amyotrophic lateral sclerosis (ALS) is a progressive fatal neurodegenerative disease affecting upper motor neurons (Betz cells in layer V of the primary motor cortex) and lower motor neurons (brainstem cranial motor nerve nuclei and spinal anterior horn cells) in the central nervous system. It is typically characterized by an adult onset with focal muscle weakness and wasting which then spreads to the rest of the body [1, 2]. Limb onset is the most common presentation with unilateral or bilateral limb weakness. Approximately 20%-30% of ALS patients will initially experience problems with speech and swallowing as a result of bulbar muscle dysfunction (bulbar onset) [1, 3]. From the genetic standpoint, nearly 15% of ALS cases are familial (inherited, fALS) with the GGGGCC hexanucleotide tandem repeat expansion in the *C9orf72* gene accounting for 20-50% of fALS cases [4]. The other most often mutated genes in fALS are *SOD1*, *TARDBP (TDP-43)*, *FUS*, *ANG,* and *OPTN* [4]. Most ALS patients do not have a family history of the disease and represent the sporadic form of ALS (sALS) which is underpinned by multiple rare genetic variants consistent with the polygenic/oligogenic nature of the disease [4, 5]. The latter, along with the patient’s age, environmental and social factors, as well as the co-existing medical conditions such as hyperlipidemia and diabetes could contribute to highly diverse clinical phenotypes of sALS [2, 4]. Therefore, treating sALS as one disease regardless of its heterogeneity could be one of the most important reasons for the failure of numerous clinical trials [1]. Hence, delineating the molecular basis and mechanisms of sALS clinical heterogeneity could provide a valuable lead for disease-modifying drug development as well as for the more efficient, personalized treatment of sALS patients and lessening a tremendous burden on their caregivers.

This article was previously presented in part as a meeting abstract at the 2023 Society for Neuroscience Annual Meeting on November 15, 2023.

## Case presentation

Body donors

Two human bodies were received through the Saint Louis University (SLU) Gift Body Program with signed informed consent from the donors.

Donor 1 (D1)

A 47-year-old female was initially referred for possible ALS. Approximately two years earlier, she fell with no significant injury. She began to experience progressive weakness in her lower extremities and falls. A year later, she was diagnosed with possible ALS. She started using a wheelchair approximately one year after symptom onset. She developed contractures in her upper extremities as well as dysarthria, dysphagia, and emotional lability of inappropriate laughing and crying. Past medical history was negative. The family history for ALS was negative. Physical examination showed a pseudobulbar effect, anarthria, facial muscle weakness, tongue fasciculation, and diffuse atrophy of limb muscles with spasticity. Reflexes were brisk. Imaging studies of the complete spine were noncontributory. Nerve conduction and electromyography were consistent with motor neuron disease. The patient developed shortness of breath and required a gastrostomy tube for nutrition. The patient passed away three years after symptom onset.

Donor 2 (D2)

A 61-year-old female began experiencing right upper extremity twitches and later noticed fasciculation in the left upper extremity. She began experiencing weakness of the left upper extremity one year prior to the presentation. Later, she developed spasms, cramps, and muscle twitches in both lower extremities. The patient did not have dysarthria, dysphagia, or shortness of breath. The family history for ALS was negative. Past medical history was significant for anxiety, depression, hyperlipidemia, osteopenia, colonic polyps, and essential tremors. Physical examination was significant for normal cranial nerves, left upper extremity motor strength of 3-4+/5 (Medical Research Council scale), atrophy of small muscles of the left hand, and brisk knee reflexes with down-going plantars. The MRI scan of the cervical spine did not show spinal stenosis. The patient underwent three nerve conduction and electromyography studies, which were consistent with motor neuron disease. Two years later, she began experiencing shortness of breath and dysphagia. She began experiencing weakness in both lower extremities. The patient sustained subdural and epidural hematomas due to a mechanical fall, from which she recovered with retrograde amnesia. The patient continued to progress and passed away 10 years after symptom onset.

Genetic analysis

The whole exome sequencing (WES) on the Next-Generation Sequencing (NGS) platform and bioinformatics analysis were performed as previously described [6, 7], with the following modifications. DNA extracted from the tibia specimens procured from the embalmed subject bodies was sequenced to a 30× depth of coverage (~4.5 Gb) on the Illumina HiSeq 2500 NGS platform in the 2 × 100 base read format. The 30× depth of coverage fulfills a requirement for the detection of human genome mutations (10× to 30×, Illumina). DNA extraction was performed by the Paleo-DNA Laboratory (Lakehead University, Canada), and the whole exome sequencing was conducted by Omega Bioservices (Norcross, GA). The cumulative exome coverage for >25× depth of coverage for D1 and D2 were 91.5% and 97.1%, respectively. The variant call and annotation were performed by the Genome Technology Access Center (GTAC, Washington University in St. Louis) using SnpSift varType and DRAGEN. The resultant data were presented in Microsoft Excel format, and rare (minor allele frequency (MAF) ≤ 0.01) pathologic/deleterious variants were identified through five consecutive filtering steps described elsewhere [6, 7]. The three final filtering steps in the bioinformatics analysis employed the SIFT [8], PolyPhen_2-HDIV [9], and PROVEAN [10] algorithms. Functional annotation of the remaining variants was performed by searching the GeneCards, Google Scholar, and PubMed databases.

Results

The WES on the Illumina NGS platform revealed that DNA procured from D1 and D2 had a set of five shared genes affected in both donors by rare mutations (MAF ≤ 0.01) that were identified through a very stringent bioinformatics analysis [6, 7] as pathologic/deleterious (Table 1). Importantly, genetic variants in three of those genes, *BBS12*, *HLA-DQB1*, and *MUC20*, were exact matches between D1 and D2 (Group I) (Table 1).

**Table 1:** Shared genes mutated in D1 and D2 sALS subjects and their respective rare pathologic/deleterious variants (Group I). Embolden text depicts genes with an exact variant match between D1 and D2. HET: heterozygous allele, MAF: minor allele frequency, sALS: sporadic amyotrophic lateral sclerosis.

Gene	Protein	Genetic Variant	MAF	Type
ACSM5	Acyl-CoA synthetase medium chain, family member 5	D1:NM_017888:exon3:c.C370T:p.R124W	0.0082	HET
		D2:NM_017888:exon11:c.C1365G:p.D455E	0.0029	HET
BBS12	Bardet-Biedl syndrome 12 protein	D1/D2:NM_001178007:exon3:c.T116C:p.I39T	0.0063	HET
HLA-DQB1	HLA class II histocompatibility antigen, DQ beta 1 chain	D1/D2:NM_002123:exon2:c.A185G:p.Y62C	0.0094	HET
		D1:NM_002123:exon2:c.T184A:p.Y62N	0.01	HET
MUC20	Mucin-20	D1/D2:NM_152673:exon2:c.G443T:p.S148I	0.0008	HET
OBSCN	Obscurin	D1:NM_001098623:exon84:c.A19297G:p.N6 33D,NM_001271223:exon95:c.A22168G:p.N7390D	8.06E-06	HET
		D2:NM_052843:exon30:c.C7981T:p.R2661C,NM_001271223:exon35:c.C9268T:p.R3090C	1.61E-05	HET

Additionally, a large number of additional relevant genes with rare pathologic/deleterious mutations in both donors were identified and grouped, based on the database and literature searches, into the following categories: variants linked to ALS (Group II), variants linked to other neurologic disorders (Group III), and variants linked to ALS-related pathology (Group IV).

Variants linked to ALS (Group II) are listed in Tables 2, 3.

**Table 2:** Genes with rare pathologic/deleterious genetic variants in D1 linked to ALS (Group II). HET: heterozygous allele, MAF: minor allele frequency, ALS: amyotrophic lateral sclerosis, sALS: sporadic amyotrophic lateral sclerosis.

Gene	Protein Function
ATP2C2	Calcium-transporting ATPase type 2C member 2. Locus functionally linked to ALS. Potential blood-based ALS biomarker.
CABIN1	Calcineurin-binding protein cabin-1. Identified as a new ALS susceptibility locus in Han Chinese.
CACNA1C	Voltage-dependent L-type calcium channel subunit alpha-1C. Identified as a causative ALS gene.
CAPNS2	Calpain small subunit 2. Identified as ALS candidate gene.
CCDC8	Coiled-coil domain-containing protein 8. Identified as a novel coding variant segregating with ALS in pedigree FALS3. Family members of the latter were tested negative for SOD1, TDP-43, FUS, ANG, ATXN2 and GRN mutations.
FER1L6	Fer-1-like protein 6. Locus functionally linked to ALS. Potential blood-based ALS biomarker.
FHDC1	FH2 domain-containing protein 1. Identified as a potential ALS risk factor. Locus functionally linked to ALS. Potential blood-based ALS biomarker.
HLA-A	HLA class I histocompatibility antigen, A alpha chain. Susceptibility to ALS.
ITGB3	Integrin beta-3. Possible ALS susceptibility gene. Upregulated in the spinal cord of ALS patients.
LRBA	Lipopolysaccharide-responsive and beige-like anchor protein. Linked to adult-onset sporadic ALS.
MAP1A	Microtubule-associated protein 1A. Suggested as a candidate gene in the autosomal recessive form of juvenile ALS (ALS5).
MAP7D2	MAP7 domain-containing protein 2. Identified as a new "switch" gene in ALS. The "switch" gene is the one that is responsible for drastic gene expression changes that may play a fundamental role in disease pathogenesis.
MEF2A	Myocyte-specific enhancer factor 2A. Potentially linked to ALS.
MYH15	Myosin-15. Muscle contraction. Its rare genetic variants in the coiled-coil tale represent a risk factor for ALS.
NEB	Nebulin. Potential ALS CSF biomarker. Most frequently mutated gene in Nemaline Myopathy.
OBSCN	Obscurin. Compound heterozygous de novo mutations with MAF < 0.01 were identified in a large cohort of ALS patient–parent trios.
PAPBC1	Polyadenylate-binding protein 1. Differentially regulated gene in sALS.
PIK3C2B	Phosphatidylinositol 4-phosphate 3-kinase C2 domain-containing subunit beta. Its novel de novo mutation p. A866T was identified in ALS.
PLEC	Plectin. Its p.R890C mutation was linked to atypical familial ALS with slowly progressing lower extremities-predominant late-onset muscular weakness and atrophy.
PTPRF	Receptor-type tyrosine-protein phosphatase F. Named as one of the 79 reported target genes related to sALS pathogenesis. Those genes include 49 susceptibility genes and 32 pathogenic genes from GWAS and OMIM.
STX2	Syntaxin 2. Associated with ALS.

**Table 3:** Genes with rare pathologic/deleterious genetic variants in D2 linked to ALS (Group II). HET: heterozygous allele, MAF: minor allele frequency, ALS: amyotrophic lateral sclerosis, sALS: sporadic amyotrophic lateral sclerosis.

Gene	Protein Function
COL28A1	Collagen Type XXVIII Alpha 1 chain. One of the chromosomal loci functionally linked to ALS.
FAM149A	Family with sequence similarity 149 member A. One of the chromosomal loci functionally linked to ALS.
LRRK1	Leucine-rich repeat kinase 1. Linked to sporadic ALS based on differential methylation in at least five ALS patients.
OBSCN	Obscurin. Compound heterozygous de novo mutations with MAF < 0.01 were identified in a large cohort of ALS patient–parent trios.
TBC1D9B	TBC1 domain family member 9B. Involved in mitochondria homeostasis regulation as well as autophagy. Therefore, could be linked to multiple neurologic disorders including ALS, Alzheimer’s, and Parkinson’s diseases.
TCF25	Transcription factor 25. Regulates ribosomal protein quality control thereby being potentially linked to ALS, Huntington's disease, and Alzheimer’s disease.
TMEM132D	Transmembrane protein 132D. One of the chromosomal loci functionally linked to ALS.
TNIP1	TNFAIP3 interacting protein 1. There is a significant association of rs10463311 spanning GPX3-TNIP1 with ALS. Associated with ALS risk. Both GPX3 and TNIP1 interact with other known ALS genes, SOD1 and OPTN. GPX3 was a lead ALS risk gene in GPX3/TNIP1 locus, with more data needed to confirm or reject a role for TNIP1.
TTN	Titin. Decreased expression of TTN is linked to a rapid functional decline in patients with sALS. Compared to health controls, ALS patients had significantly increased levels of titin-N-terminal fragment normalized to creatine (Cr) level. It was correlated with the revised ALS functional rating scale. The urinary titin/Cr and serum neurofilament levels were independent factors for a poor prognosis.

Variants linked to other neurologic disorders (Group III) are listed in Tables 4, 5.

**Table 4:** Genes with rare pathologic/deleterious genetic variants in D1 linked to other neurologic disorders (Group III). HET: heterozygous allele, MAF: minor allele frequency, ALS: amyotrophic lateral sclerosis, sALS: sporadic amyotrophic lateral sclerosis.

Gene	Protein Function
ATCAY	Caytaxin. Cayman-type cerebellar ataxia (PMID: 17303531) and dystonia (PMID: 17303531; PMID: 18285800).
BBS12	Bardet–Biedl syndrome 12 protein. Bardet–Biedl syndrome 12 and Bardet–Biedl syndrome (PMID: 17160889).
DBX2	Homeobox protein DBX2. Miles–Carpenter syndrome.
DNAJC13	DnaJ homolog subfamily C member 13. Hereditary late-onset Parkinson’s disease and essential tremor.
DOCK7	Dedicator of cytokinesis protein 7. Mutations in DOCK7 were found in individuals with epileptic encephalopathy and cortical blindness.
FER1L6	Fer-1-like protein 6. Miyoshi muscular dystrophy and deafness, autosomal recessive 9.
HLA-A	HLA class I histocompatibility antigen, A alpha chain. Susceptibility to multiple sclerosis, Alzheimer’s disease, and schizophrenia.
*HLA-DQB*1	HLA class II histocompatibility antigen, DR beta 5 chain. Susceptibility to myasthenia gravis and multiple sclerosis. Inherited Creutzfeldt–Jakob disease.
ITGA8	Integrin alpha-8. A risk gene locus for Parkinson's disease.
MEF2A	Myocyte-specific enhancer factor 2A. Potentially linked to multiple sclerosis. Linked to Alzheimer’s disease.
NEB	Nebulin. The most frequently mutated gene in nemaline myopathy.
NGLY1	Peptide-N(4)-(N-acetyl-beta-glucosaminyl) asparagine amidase. A homozygous frameshift mutation caused neuromotor impairment, apparent intellectual disability, corneal opacity, and neuropathy in two siblings. Was not expressed in the majority of neurons of C9FTD/ALS brain sections compared to non-demented controls.
OBSCN	Obscurin. Was among 180 genes associated with primary myopathies.
PDE4D	cAMP-specific 3',5'-cyclic phosphodiesterase 4D. A risk factor for Alzheimer’s disease and other neural degenerative diseases. Associated with nicotine dependence.
RBMX	RNA-binding motif protein, X chromosome. Component of the DNA damage response. Key modulator of alternative splicing in multiple genes implicated in neurodegenerative diseases including SMN2 in spinal muscular atrophy and microtubule-associated protein tau (MAPT) in FTLD. May be involved in the negative regulation of proximal spinal muscular atrophy. Syndromic X-linked intellectual disability, Shashi type.
SEZ6L2	Seizure 6-like protein 2. Its antibody was associated with acute cerebellar ataxia.
SLC6A1	Sodium- and chloride-dependent GABA transporter 1 (GAT1). Associated with myoclonic-atonic epilepsy. Linked to epilepsy, schizophrenia, and anxiety. Genetic variation within the *GAT1* gene may be associated with anxiety disorders with panic syndrome. GAT1 inhibition for the treatment of seizures leads to side effects such as fatigue, dizziness, psychomotor slowing, ataxia, GI upset, weight change, and insomnia.
SNX17	Sorting nexin-17. Linked to an autosomal-dominant form of neurodegeneration. Associated with hypercholesterolemia, familial, 4.
STX2	Syntaxin 2. Associated with Alzheimer’s and Parkinson’s diseases.
TBC1D2	TBC1 domain family member 2A. The mutations in TBC1D2 cause focal epilepsy. Associated with pontocerebellar hypoplasia, Warburg micro syndrome 1.
WDFY3	WD repeat and FYVE domain-containing protein 3 (autophagy-linked FYVE protein). Autism. A spectrum of WDFY3 is neurodevelopmental delay, intellectual disability, macrocephaly, and psychiatric disorders (autism spectrum disorders/attention deficit hyperactivity disorder).
ZBTB10	Zinc finger and BTB domain-containing protein 10. A novel candidate gene linked to myasthenia gravis, late-onset. Differentially regulated gene in Huntington's disease.

**Table 5:** Genes with rare pathologic/deleterious genetic variants in D2 linked to other neurologic disorders (Group III). ALS: amyotrophic lateral sclerosis.

Gene	Protein Function
AHNAK2	AHNAK nucleoprotein 2. The causative gene for autosomal recessive axonal and/or demyelinating form of Charcot–Marie–Tooth disease. Is a component of the dysferlin protein complex and could be associated with dysferlinopathies – a muscle disease group that includes Miyoshi muscular dystrophy and limb-girdle muscular dystrophy type 2B. Developmental and epileptic encephalopathy 66.
ASB3;GPR75	Ankyrin repeat and SOCS box containing 3. Neuronitis.
BBS12	Bardet–Biedl syndrome 12 protein. Bardet–Biedl syndrome 12 and Bardet–Biedl syndrome.
BPGM	Bisphosphoglycerate mutase. Differentially regulated gene in Parkinson’s disease.
C1orf185	Chromosome 1 open reading frame 185. Charcot–Marie–Tooth disease, recessive intermediate B.
C9	Complement component C9. In large multiple sclerosis lesions, C1q-C9 immunoreactive fibers were present. A direct attack on oligodendrocyte cells by the early complement components could be an initiating event in multiple sclerosis.
CCDC136	Coiled-coil domain containing protein 136. Dyscalculia and reading disorder. Could be associated with hypoxic-ischemic encephalopathy (DOI: 10.1002/ibra.12025). Protein with post-translational modification differentially expressed in Alzheimer’s disease.
CDHR2	Cadherin-related family member 2. Usher syndrome, Type I.
CHD4	Chromodomain-helicase-DNA-binding protein 4. Sifrim–Hitz–Weiss syndrome and Helsmoortel–Van Der Aa Syndrome. Associated with an autosomal dominant intellectual disability syndrome with features and affected systems including cardiac, skeletal, and urogenital. The subject with a CHD4 variant had seizures and a diagnosis of Landau–Kleffner syndrome.
COL28A1	Collagen type XXVIII alpha 1 chain. Cerebellar ataxia type 9. Its rs6962939 SNV was linked to frontotemporal lobar degeneration (FTLD). Protein was downregulated by ~3-fold in the brain of a mouse model of Alzheimer’s disease.
DST	Dystonin; bullous pemphigoid antigen 1 (BPAG1). Neuropathy, hereditary sensory and autonomic, type VI. The absence of the N-terminus domain of the neuronal BPAG1 (BPAG1n) results in dystonia musculorum in mice. Bullous pemphigoid (BP) occurs in many patients with multiple sclerosis and a small number of ALS patients. The mean age of three patients at the onset of BP was 52 years, which is substantially lower than the average age of BP onset. Light and EM alterations of skin connective tissue have recently been described in seven patients with ALS, with cutaneous deposition of β-amyloid protein.
ECE1	Endothelin-converting enzyme 1. Hirschsprung disease. Potentially disease-modifying gene in Parkinson’s disease.
FAM149A	Family with sequence similarity 149 member A. Facioscapulohumeral muscular dystrophy 1 and Waardenburg syndrome, Type 3.
FAT2	FAT atypical cadherin 2. Spinocerebellar ataxia 45 and spinocerebellar ataxia 40 and spinocerebellar ataxia.
FBN3	Fibrillin-3. Involved in pathogenesis of a Chinese family with Bardet–Biedl syndrome.
HLA-DQB1	Major histocompatibility complex, Class II, DQ beta 1. Susceptibility to myasthenia gravis and multiple sclerosis. Inherited Creutzfeldt–Jakob disease.
LRRK1	Leucine-rich repeat kinase 1. Parkinson’s disease.
MYO7A	Myosin VIIA. Usher syndrome, Type I and deafness, autosomal dominant 11. Autosomal recessive nonsyndromic hearing loss 2. Usher syndrome.
PAPLN	Papilin, proteoglycan-like sulfated glycoprotein. Radial nerve lesion. Non-syndromic X-linked intellectual disability 2.
PDE8B	Phosphodiesterase 8B. Striatal degeneration, autosomal dominant 1. Pigmented nodular adrenocortical disease, primary, 3, and striatal degeneration, autosomal dominant 1.
PIK3C3	Phosphatidylinositol 3-kinase catalytic subunit type 3. Neurodegeneration with brain iron accumulation and myopathy, X-linked, with excessive autophagy.
PLCXD1	Phosphatidylinositol-specific phospholipase C X domain containing 1. Downregulated gene in Parkinson’s disease.
PRAMEF10	PRAME family member 10. Dandy–Walker syndrome.
RBFA	Ribosome-binding factor A. DYT1 dystonia (N245H variant, bioRxiv). Copy number variation was linked to autism spectrum disorder.
RILP	Rab interacting lysosomal protein. Charcot–Marie–Tooth disease, axonal, Type 2B and Charcot–Marie–Tooth disease, axonal, Type 2E.
SLC25A47	Solute carrier family 25 member 47. Early infantile epileptic encephalopathy.
SLC52A1	Solute carrier family 52 member 1. Brown–Vialetto–Van Laere syndrome (PMID: 23107375). Multiple acyl-CoA dehydrogenase deficiency disorder.
TBC1D9B	TBC1 domain family member 9B. Involved in mitochondria homeostasis regulation as well as autophagy. Therefore, could be linked to multiple neurologic disorders including ALS, Alzheimer’s, and Parkinson’s diseases.
TCF25	Transcription factor 25. Regulates ribosomal protein quality control thereby being potentially linked to ALS, Huntington's disease, and Alzheimer’s disease.
TIMM44	Translocase of inner mitochondrial membrane 44. Spinocerebellar ataxia 28 and developmental and epileptic encephalopathy 12.
TM2D3	TM2 domain containing 3. Alzheimer’s disease, late-onset (PMID: 27764101). Alzheimer’s disease. Variants of uncertain significance linked to dementia, early onset.
TMEM132D	Transmembrane protein 132D. Spinocerebellar ataxia 23 and panic disorder. The protein level in CSF was downregulated in patients with the behavioral variant of frontotemporal dementia (bvFTD) as compared to non-carriers. The rs660322 variant is linked to the rate of cognitive decline in patients with Alzheimer’s disease. Potentially associated with panic syndrome. High TMEM132D expression is associated with increased anxiety levels.
TMEM243	Transmembrane protein 243. Upregulated in the substantia nigra and blood of Parkinson’s disease patients. Can be considered as a Parkinson’s disease biomarker.
TRIM54	Tripartite motif containing 54 (MuRF3). Distal myopathy, myofibrillar myopathy, and restrictive cardiomyopathy-5. Hyaline body myopathy and myopathy, myofibrillar, 9, with early respiratory failure. Cardiac and skeletal aggregate myopathy.
TTN	Titin. Myopathy, myofibrillar, 9, with early respiratory failure and congenital myopathy 5 with cardiomyopathy.
USH2A	Usherin. Usher syndrome, Type IIa.
VCPKMT	Valosin containing protein lysine methyltransferase. Pontocerebellar hypoplasia, Type 2A.
VCX3A	Variable charge X-linked 3A. Gene copy number variation is linked to Rolandic epilepsy.
ZNF536	Zinc finger protein 536. One of the risk genes for neurodegeneration with brain iron accumulation.

Variants linked to ALS-related pathology (Group IV) are listed in Tables 6, 7.

**Table 6:** Genes with rare pathologic/deleterious variants in D1 linked to ALS-related pathology (Group IV). ALS: amyotrophic lateral sclerosis.

Gene	Protein Function
DOCK7	Dedicator of cytokinesis protein 7. Regulates axon formation and myelination. Regulates cortical neurogenesis through its expression in chandelier cells.
MAP7D2	MAP7 domain-containing protein 2. Involved in microtubule cytoskeleton organization. Identified as a new "switch" gene in ALS. The "switch" gene is the one that is responsible for drastic gene expression changes that may play a fundamental role in disease pathogenesis.
MEF2A	Myocyte-specific enhancer factor 2A. Transcription factor. Required for skeletal muscle regeneration. Microglia homeostatic gene. Enriched in microglia from SOD1 (G93A) mice (ALS model). Activated by TGF to induce tolerogenic phenotype of microglia and is suppressed by Apo E activation to induce microglia neurodegenerative phenotype. Therefore, TGF activates, and Apo E suppresses the MEF2A-dependent transcriptional program in microglia. This microglia Apo E-dependent switch is present in ALS, MS, and AD.
NEB	Nebulin. A significantly downregulated gene in skeletal muscles indicating the decreased length of thin filaments in ALS patients.
PABPC1	Polyadenylate-binding protein 1. Central stress granule component in the spinal cord and motor neurons. In Drosophila, its co-expression with either TDP-43 or ATXN2-32Q enhanced toxicity, resulting in more severe retinal degeneration. Interaction of PABPC1 with TDP-43 or altered PABPC1 function might occur in ALS producing punctate structures in the spinal cord. One of the c9orf72 hexanucleotide repeat binding proteins with RRM motifs.
PPARGC1A	Peroxisome proliferator-activated receptor gamma coactivator 1-alpha. Deficiency in full-length PGC-1a leads to a significantly earlier age of onset and a borderline shortened survival in male, but not in female transgenic ALS mice. Overexpression of PPARGC1A in SOD1 transgenic mice (murine ALS model) significantly improved motor function and survival of experimental animals.
PRIMPOL	DNA-directed primase/polymerase protein. Mediates DNA-RNA hybrid homeostasis by regulating R-loop impaired replication. TDP-43 pathology is linked to increased R-loops and R-loop-mediated DNA damage. The role of DNA damage in the pathogenesis of C9orf72 repeat expansion is emerging.
PRR7	Proline-rich protein 7. Induces specific removal of excitatory synapses and acts as a Wnt inhibitor. Released from exosomes in an activity-dependent manner and eliminates excitatory synapses in neighboring neurons following their uptake via membrane fusion. Extracellular vesicles (exosomes and ectosomes) play key roles in the pathology of brain diseases.
PTBP3	Polypyrimidine tract-binding protein 3. RNA-binding protein that mediates pre-mRNA alternative splicing regulation. Plays a role in the regulation of cell proliferation, differentiation, and migration. Interacts with TDP-43 in spinal cord lysates from control subjects. Plays a role in nonsense-mediated mRNA decay. Premature termination codons in SOD1 causing ALS are predicted to escape the nonsense-mediated mRNA decay.
RBMX	RNA-binding motif protein, X chromosome. Component of the DNA damage response. Key modulator of alternative splicing in multiple genes implicated in neurodegenerative diseases including SMN2 in spinal muscular atrophy and microtubule-associated protein tau in frontotemporal lobar degeneration. Identified as a novel splicing factor that promotes inclusion of SMN2 exon 7 leading to an increase in the endogenous SMN2 levels. The latter could partially compensate for the homozygous loss of SMN1 in proximal spinal muscular atrophy.
SIK2	Serine/threonine-protein kinase SIK2. SIK2 and its kinase activity are indispensable for the removal of TDP-43 inclusion bodies making this protein a critical determinant of the autophagy progression.
SNX17	Sorting nexin-17. Identified as one of six genes that have variants causing autosomal dominant forms of neurodegeneration and serving as genetic regulators of microglial lipid droplet formation. The lipid droplet-associated microglia (LDAM) has been recently identified as a novel state of microglia in the aging brain.
USP15	Ubiquitin carboxyl-terminal hydrolase 15. Lack of the USP15 gene induces an impairment in motor ability in an age-dependent manner with an unconventional cerebellar formation.
WDFY3	WD repeat and FYVE domain-containing protein 3 (autophagy-linked FYVE protein). Promotes autophagic removal of misfolded proteins involved in ALS. The clearance of intracellular SOD1, TDP-43, and its C-terminal fragment TDP-25 aggregates is dependent on WDFY3. Required for sustaining brain bioenergetics and morphology via mitophagy. Wdfy3 haploinsufficiency in mice results in decreased mitophagy and mitochondrial localization at synaptic terminals and decreased synaptic density that may contribute to altered synaptic plasticity. WDFY3 impairment leads to age-dependent formation of brain glycogen deposits and cerebellar hypoplasia. A spectrum of WDFY3 is linked to neurodevelopmental delay in humans and intellectual disability.

**Table 7:** Genes with rare pathologic/deleterious genetic variants in D2 linked to ALS-related pathology (Group IV). ALS: amyotrophic lateral sclerosis

Gene	Protein Function
APOA5	Apolipoprotein A5. Hypertriglyceridemia 1 and hyperlipoproteinemia, type V. Patients with elevated triglyceride and cholesterol serum levels have a prolonged survival in ALS.
ARID4B	AT-rich interactive domain-containing protein 4B. One of three genes identified in drosophila (hat-trick) that suppress age-dependent TDP-43-mediated motor neuron degeneration.
BPGM	Bisphosphoglycerate mutase. Downregulated ~2.5-fold in the skeletal muscle of 24-week-old SOD1 mice (ALS model) as compared to control wild-type mice.
CCDC136	Coiled-coil domain-containing protein 136. The CCDC136/FLNC locus is associated with reading and language traits.
CHD1	Chromodomain-helicase-DNA-binding protein 1. TDP-43-induced toxicity in the Drosophila model of ALS was strongly enhanced by CHD1 knockdown. Physically interacts with TDP-43 protein.
DCAF4	DDB1- and CUL4-associated factor 4. Its mediated ubiquitination of optineurin stimulates autophagic degradation of Cu, Zn-superoxide dismutase.
ECE1	Endothelin-converting enzyme 1. ECE1 converts endothelin-1 precursor into bioactive peptide. Expression of ECE1 in vivo suppressed dopamine neuron loss and alleviated the corresponding motor deficits in mice with α-synuclein A3OP expression (PMID: 29524599). Endothelin-1 is overexpressed in ALS and induces motor neuron death.
ENPP2	Ectonucleotide pyrophosphatase/phosphodiesterase 2 (Autotaxin, ATX). ATX is a potential target and/or biomarker in ALS and highlights ATX inhibitors as a potentially reasonable tool for the treatment of ALS.
FAT4	FAT atypical cadherin 4. Mutations in genes encoding the cadherin–receptor pair FAT4-DCHS disrupt cerebral cortical development.
FRMD1	FERM domain containing 1. Predicted to be involved in the positive regulation of Hippo signaling. Hyperactivation of the Hippo pathway is linked to ALS and Alzheimer’s disease. MST1 (human Hippo homolog) is a key modulator of neurodegeneration in a mouse model of ALS.
IL17RA	Interleukin 17 receptor A. Th17 cells and IL-17A directly contribute to motor neuron degeneration.
KIR3DS1	Killer cell immunoglobulin-like receptor, three Ig domains, and short cytoplasmic tail 1. HLA-F was identified as a KIR3DS1 ligand. HLA-F was found to contribute to ALS pathogenesis: Its overexpression protects human motor neurons from toxicity mediated by ALS astrocytes.
MAB21L3	Mab-21 like 3. Loss of function causes a syndromic neurodevelopmental disorder with distinctive cerebellar, ocular, craniofacial, and genital features (COFG syndrome).
MYH2	Myosin heavy chain 2. Protein levels were significantly increased in the sera from patients with ALS behavioral variant of frontotemporal dementia (PMID: 32792518). There was a 3-fold decrease in gene expression in myotubes from mice with SOD1 (G93A) mutation.
MYH9	Myosin heavy chain 9. Overexpression of TDP-43 in rat cortical neurons upregulated MYH9 expression by ~2-fold. In neurons, MYH9 is important for driving neurite outgrowth, is involved in growth cone motility, and regulates NMDA receptor trafficking. Alterations in MYH9 protein expression levels have been found in both the ALS patients' brains containing TDP-43 aggregates as well as in transgenic pigs overexpressing the TDP-43 M337V mutation.
NOX5	NADPH oxidase 5. Genetic deletion of NOX isoforms is neuroprotective in mouse models of PD, stroke, and prion disease. Upregulated NOX5 expression and downregulated levels of NOX4 in serum from MS patients might be related to vascular changes in the setting of oxidative stress.
PCDHA1-12	Protocadherin alpha-11. PCDHA knockout mice had changes in interneurons in the prefrontal cortex, including a decreased number of neurites from the soma, decreased total branch number, and decreased neurite length. A similar pattern was observed in iPS-derived cortical interneurons from subjects with schizophrenia.
PDGFRA	Platelet-derived growth factor receptor alpha. PDGFRA/NG2 glia generate myelinating oligodendrocytes and piriform projection neurons in mice.
PIEZO1	Piezo-type mechanosensitive ion channel component 1. Acts as a sensor of phosphatidylserine (PS) flipping at the plasma membrane and governs the morphogenesis of muscle cells.
PTPRZ1	Protein tyrosine phosphatase receptor type Z1. Astrocyte-specific gene. Protein abundance was changed ~2-fold in exome-enriched fractions from CSF of ALS patients.
RAB11FIP1	RAB11 family interacting protein 1. Upregulated gene in the spinal cord of ALS patients.
RETSAT	Retinol saturase. Protein was identified as a part of the natural killer (NK) cell membrane proteome. Expression was upregulated by ~3.4-fold in the fetal spinal cord at embryonic day 17 in rats with myelomeningocele induced by retinoic acid.
RRBP1	Ribosome binding protein 1. Inhibited by miR-206 with important implications for muscle regeneration. Interacts with TDP-43 protein.
SLC22A13	Solute carrier family 22 member 13. One of the microsatellite markers found in ALS patients. In the 400 kb region between two markers, there were eight genes, including SLC22A13.
SLCO2A1	Solute carrier organic anion transporter family member 2A1. Differentially regulated (downregulated) gene the choroid plexus of ALS patients.
TJP1	Tight junction protein 1 (ZO-1). A significant reduction in the expression levels of tight junction protein ZO-1 correlated with the tight junction damage and increased permeability was found in the SOD1G93A mouse model of ALS. Downregulated in the spinal cord of mice with experimental autoimmune encephalomyelitis, an animal model of multiple sclerosis.
ZDHHC11	Zinc finger DHHC-type containing 11. Downregulated in the frontal cortex of ALS patients.

**Figure 1:**
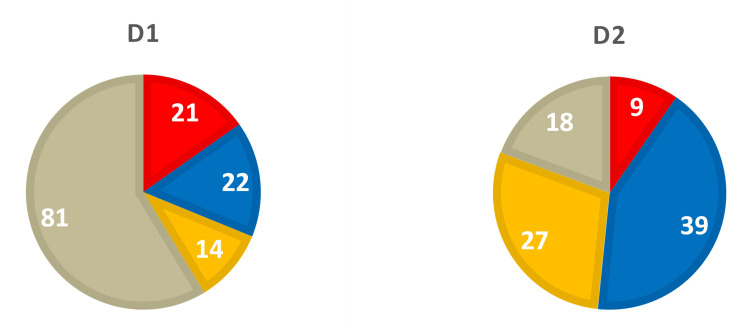
Distribution of rare pathologic/deleterious variants in D1 and D2 among the following categories: red - variants linked to amyotrophic lateral sclerosis (ALS) (Group II); blue - variants linked to other neurological disorders (Group III); yellow - variants linked to ALS-related pathology (Group IV); and beige - ungrouped variants. Numerals represent the total number of variants in the respective categories.

Analysis of the specific groups presented in those tables revealed significant differences between D1 and D2. Specifically, D1, with early bulbar onset and fast sporadic ALS (sALS) progression, had 21 mutated genes in Group II (Table 2) and 22 genes in Group III, including two genes linked to Myasthenia Gravis (MG) (HLA-DQB1 and ZBTB10) (Table 4). In contrast, D2, with late limb onset and slow sALS progression, had only nine variants in Group II (Table 3), 39 mutated genes in Group III, including one gene linked to MG (HLA-DQB1), along with three genes linked to Charcot-Marie-Tooth (CMT) disease (AHNAK2, C1orf185, and RILP), and a homozygous mutation in the causative gene for the autosomal recessive form of CMT disease, AHNAK2 [11] (Table 5). There were 14 and 27 mutated genes, respectively, for D1 (Table 6) and D2 (Table 7) in Group IV. The cumulative distribution of genetic variants between Groups II-IV for both donors is presented in Figure 1. As seen from this Figure, only the number of variants linked to ALS in D1 and D2 (21 vs 9) appears to correlate with the severity of their respective clinical phenotypes.

## Discussion

The genetic screen (WES) of the two sALS subjects with contrasting clinical phenotypes provided two lines of novel information that are very important for our understanding of disease development and heterogeneity. First, there was a presence of five genes that were mutated in both subjects, with three of those genes, *BBS12*, *HLA-DQB1*, and *MUC20*, characterized by identical rare pathologic/deleterious genetic variations (Table 1). Neither BBS12, HLA-DQB1, nor MUC20 have been previously linked to ALS, but they participate in important biological pathways potentially perturbed in ALS. Indeed, BBS12 is one of the ciliopathy-related genes that differentially modulate neuronal differentiation in the cerebral cortex [12]. HLA-DQB1 belongs to the human major histocompatibility gene (MHC) family and, by its participation in adaptive immunity, may elicit neuroprotection in the CNS [13]; it is also linked to neurological disorders such as MG and multiple sclerosis (MS) [14]. MUC20, expressed in the brain [15], regulates hepatocyte growth factor (HGF)/c-Met signaling [16, 17], which promotes motor neuron survival by synergizing with ciliary neurotrophic factor (CNTF) [18].

The other two genes mutated in both subjects, but with mismatched rare pathologic/deleterious genetic variants, were *ACSM5* and *OBSCN*. *ACSM5* encodes a mitochondrial enzyme, acyl-coenzyme A synthetase, which catalyzes the activation of medium-chain length fatty acids by coenzyme A (CoA), to produce an acyl-CoA, the initial step in fatty acid metabolism. *ACSM5* is mostly expressed in the liver and adipose tissue, where it plays an essential role in energy storage and metabolism [19]. Heterozygous copy number variation due to a deletion of exons 13 and 14 of *ACSM5* has been associated with dyslipidemia in humans [20]. A single-nucleotide polymorphism in *ACSM5* has also been linked to altered intramuscular fat content and its fatty acid composition in pigs [21]. Therefore, it would be reasonable to suggest that altered *ACSM5 *function could be linked to dysregulated energy metabolism in ALS patients, where hypermetabolism has been reported as one of ALS's distinct phenotypical features [22, 23]. *OBSCN* was the only shared gene between the two subjects that has been previously associated with ALS but in a rather uncommon manner. Compound heterozygous de novo mutations with MAF < 0.01 were identified in the two ALS patient-parent trios: p.A7260T (inherited from the father) and p.R1361 (inherited from the mother); p.R5515C (inherited from the father) and p.R5920H (inherited from the mother) [24]. None of these variants were present in the current study. Yet *OBSCN* encodes a structural component of striated muscles and plays an important role in myofibrillogenesis [25] as well as in the development of the heart, skeletal muscle, and brain [26]. Most likely, by virtue of being an important muscle structural component, *OBSCN *has been also associated with skeletal muscle atrophy [27] and primary myopathy [28].

What is the meaning of the presence of the shared mutated genes in two sALS subjects with contrasting clinical phenotypes and their relevance to the disease initiation and progression? We hypothesize that based on a close link between those genes and major physiological domains perturbed in ALS - neuronal development and motor neuron survival (*BBS12, MUC20, OBSCN*), adaptive immunity (*HLA-DQB1*), skeletal muscle development and function (*OBSCN*), as well as energy metabolism (*ACSM5*) - the respective heterozygous mutations due to their presumed low penetrance would prime the individuals for sALS without its initiation. The disease in the primed individuals will then be triggered by mutations in the genes associated with ALS (Tables 2, 3) The exact type and number of genes involved in the priming step could vary, but they still must be linked to the same major physiological domains perturbed in ALS as described above.

The second line of important information obtained during the study and pertinent to sALS development and propagation is that the genetic data presented above indicated a possible contribution from other neurologic disorders, including those of MG and CMT, to the ALS clinical presentation (Tables 4, 5). Indeed, as an extreme case of such contribution, the co-occurrence of ALS and MG has been previously reported in several publications [29-33] and was shown statistically not to be coincidental [32], highlighting possible common pathologic changes in the adaptive immune response [32], affecting, most likely, the complement cascade [34]. Importantly, similar to the clinical presentation of D1, patients with co-occurring ALS and MG more frequently had bulbar onset ALS with a fast-progressing course [31]. Also important, the MG form with the auto-antibody against muscle-specific kinase (MuSK) could alone mimic bulbar onset ALS [35]. The co-occurrence of ALS with CMT disease has also been reported where slow-progressing CMT started first then followed by fast-progressing ALS [36]. It is quite possible that a large number of mixed ALS/CMT cases could have been left unnoticed due to similarities in ALS and CMT clinical phenotypes [37], attributed to common genetic underpinnings [37-39].

## Conclusions

Altogether, our data are consistent with the hypothesis of sALS development and heterogeneity, where the Group I genes could prime an individual for ALS, the Group II genes could trigger the disease, and genes from Groups III and IV would specify further the disease's pathologic components and its clinical phenotype. This hypothesis provides a novel mechanistic approach to our understanding of the sALS etiology and heterogeneity that should bestow novel venues for delivering more efficient and personalized treatment for sALS patients, as well as identifying new highly promising leads for the development of disease-modifying drugs.

## References

[1] Masrori P, Van Damme P (2020). Amyotrophic lateral sclerosis: a clinical review. Eur J Neurol.

[2] Feldman EL, Goutman SA, Petri S, Mazzini L, Savelieff MG, Shaw PJ, Sobue G (2022). Amyotrophic lateral sclerosis. Lancet.

[3] Nijssen J, Comley LH, Hedlund E (2017). Motor neuron vulnerability and resistance in amyotrophic lateral sclerosis. Acta Neuropathol.

[4] Kiernan MC, Vucic S, Talbot K (2021). Improving clinical trial outcomes in amyotrophic lateral sclerosis. Nat Rev Neurol.

[5] McCann EP, Henden L, Fifita JA (2020). Evidence for polygenic and oligogenic basis of Australian sporadic amyotrophic lateral sclerosis. J Med Genet.

[6] Jenkins M, Frolov A, Tan Y, Daly D, Lawson C, Martin JR (2019). Situs inversus totalis in a 96-year-old female cadaver: evidence pointing toward the two-cilia model. It J Anat Embryol.

[7] Frolov A, Lawson C, Olatunde J, Goodrich JT, Martin Iii JR (2019). Sagittal craniosynostosis with uncommon anatomical pathologies in a 56-year-old male cadaver. Case Rep Pathol.

[8] Sim NL, Kumar P, Hu J, Henikoff S, Schneider G, Ng PC (2012). SIFT web server: predicting effects of amino acid substitutions on proteins. Nucleic Acids Res.

[9] Adzhubei IA, Schmidt S, Peshkin L (2010). A method and server for predicting damaging missense mutations. Nat Methods.

[10] Choi Y, Chan AP (2015). PROVEAN web server: a tool to predict the functional effect of amino acid substitutions and indels. Bioinformatics.

[11] Tey S, Shahrizaila N, Drew AP (2019). Linkage analysis and whole exome sequencing reveals AHNAK2 as a novel genetic cause for autosomal recessive CMT in a Malaysian family. Neurogenetics.

[12] Guo J, Higginbotham H, Li J, Nichols J, Hirt J, Ghukasyan V, Anton ES (2015). Developmental disruptions underlying brain abnormalities in ciliopathies. Nat Commun.

[13] Béland LC, Markovinovic A, Jakovac H (2020). Immunity in amyotrophic lateral sclerosis: blurred lines between excessive inflammation and inefficient immune responses. Brain Commun.

[14] Misra MK, Damotte V, Hollenbach JA (2018). The immunogenetics of neurological disease. Immunology.

[15] Biancalani T, Scalia G, Buffoni L (2021). Deep learning and alignment of spatially resolved single-cell transcriptomes with Tangram. Nat Methods.

[16] Higuchi T, Orita T, Katsuya K (2004). MUC20 suppresses the hepatocyte growth factor-induced Grb2-Ras pathway by binding to a multifunctional docking site of met. Mol Cell Biol.

[17] Chen ST, Kuo TC, Liao YY, Lin MC, Tien YW, Huang MC (2018). Silencing of MUC20 suppresses the malignant character of pancreatic ductal adenocarcinoma cells through inhibition of the HGF/MET pathway. Oncogene.

[18] Wong V, Glass DJ, Arriaga R, Yancopoulos GD, Lindsay RM, Conn G (1997). Hepatocyte growth factor promotes motor neuron survival and synergizes with ciliary neurotrophic factor. J Biol Chem.

[19] Namjou B, Lingren T, Huang Y (2019). GWAS and enrichment analyses of non-alcoholic fatty liver disease identify new trait-associated genes and pathways across eMERGE Network. BMC Med.

[20] Marmontel O, Rollat-Farnier PA, Wozny AS (2020). Development of a new expanded next-generation sequencing panel for genetic diseases involved in dyslipidemia. Clin Genet.

[21] Puig-Oliveras A, Revilla M, Castelló A, Fernández AI, Folch JM, Ballester M (2016). Expression-based GWAS identifies variants, gene interactions and key regulators affecting intramuscular fatty acid content and composition in porcine meat. Sci Rep.

[22] Dupuis L, Pradat PF, Ludolph AC (2011). Energy metabolism in amyotrophic lateral sclerosis. Lancet Neurol.

[23] González De Aguilar JL (2019). Lipid biomarkers for amyotrophic lateral sclerosis. Front Neurol.

[24] van Doormaal PT, Ticozzi N, Weishaupt JH (2017). The role of de novo mutations in the development of amyotrophic lateral sclerosis. Hum Mutat.

[25] Perry NA, Ackermann MA, Shriver M, Hu LY, Kontrogianni-Konstantopoulos A (2013). Obscurins: unassuming giants enter the spotlight. IUBMB Life.

[26] Raeker MO, Bieniek AN, Ryan AS, Tsai HJ, Zahn KM, Russell MW (2010). Targeted deletion of the zebrafish obscurin A RhoGEF domain affects heart, skeletal muscle and brain development. Dev Biol.

[27] Qiu J, Wu L, Chang Y, Sun H, Sun J (2021). Alternative splicing transitions associate with emerging atrophy phenotype during denervation-induced skeletal muscle atrophy. J Cell Physiol.

[28] Evilä A, Arumilli M, Udd B, Hackman P (2016). Targeted next-generation sequencing assay for detection of mutations in primary myopathies. Neuromuscul Disord.

[29] Cho EB, Yang TW, Jeong H (2019). Uncommon coexistence of myasthenia gravis and amyotrophic lateral sclerosis. Ann Clin Neurophysiol.

[30] Tai H, Cui L, Guan Y (2017). Amyotrophic lateral sclerosis and myasthenia gravis overlap syndrome: a review of two cases and the associated literature. Front Neurol.

[31] de Pasqua S, Cavallieri F, D'Angelo R (2017). Amyotrophic lateral sclerosis and myasthenia gravis: association or chance occurrence?. Neurol Sci.

[32] Del Mar Amador M, Vandenberghe N, Berhoune N (2016). Unusual association of amyotrophic lateral sclerosis and myasthenia gravis: a dysregulation of the adaptive immune system?. Neuromuscul Disord.

[33] Mulder DW, Lambert EH, Eaton LM (1959). Myasthenic syndrome in patients with amyotrophic lateral sclerosis. Neurology.

[34] Lee JD, Woodruff TM (2021). The emerging role of complement in neuromuscular disorders. Semin Immunopathol.

[35] Gilhus NE, Verschuuren JJ (2015). Myasthenia gravis: subgroup classification and therapeutic strategies. Lancet Neurol.

[36] Marchesi C, Ciano C, Salsano E (2011). Co-occurrence of amyotrophic lateral sclerosis and Charcot-Marie-Tooth disease type 2A in a patient with a novel mutation in the mitofusin-2 gene. Neuromuscul Disord.

[37] Martin PB, Hicks AN, Holbrook SE, Cox GA (2020). Overlapping spectrums: the clinicogenetic commonalities between Charcot-Marie-Tooth and other neurodegenerative diseases. Brain Res.

[38] Montecchiani C, Pedace L, Lo Giudice T (2016). ALS5/SPG11/KIAA1840 mutations cause autosomal recessive axonal Charcot-Marie-Tooth disease. Brain.

[39] He J, Liu X, Tang L, Zhao C, He J, Fan D (2020). Whole-exome sequencing identified novel KIF5A mutations in Chinese patients with amyotrophic lateral sclerosis and Charcot-Marie-Tooth type 2. J Neurol Neurosurg Psychiatry.

